# Exploitation of *Vitis vinifera*, *Foeniculum vulgare*, *Cannabis sativa* and *Punica granatum* By-Product Seeds as Dermo-Cosmetic Agents

**DOI:** 10.3390/molecules26030731

**Published:** 2021-01-31

**Authors:** Dimitris Michailidis, Apostolis Angelis, Panagiota Efstathia Nikolaou, Sofia Mitakou, Alexios Léandros Skaltsounis

**Affiliations:** 1 Faculty of Pharmacy, Department of Pharmacognosy and Natural Products Chemistry, National and Kapodistrian University of Athens, 15772 Athens, Greece; dmichail@pharm.uoa.gr (D.M.); aangjel@pharm.uoa.gr (A.A.); mitakou@pharm.uoa.gr (S.M.); 2 Faculty of Pharmacy, Department of Pharmacology, National and Kapodistrian University of Athens, 15771 Athens, Greece; nayanik@pharm.uoa.gr

**Keywords:** grapevine, fennel, hemp, pomegranate, seed by-products, supercritical fluid extraction, ultrasound extraction, anti-tyrosinase, anti-collagenase, anti-elastase

## Abstract

In the current study, by-product seed pastes (VSPs) from *Vitis vinifera*, *Foeniculum vulgare*, *Cannabis sativa* and *Punica granatum*, generated during the oil production process, were investigated for their potential exploitation as dermo-cosmetic agent. The extraction pipeline of all the raw materials was developed with emphasis on green methodologies and employed on laboratory scale based on industry-adopted techniques. Two different protocols were applied, Supercritical Fluid Extraction (SFE) and Ultrasound Assisted Extraction (UAE); the by-product pastes were defatted with supercritical CO_2_ and *n-*Hexane, respectively. Then, two SFE extracts (CO_2_ with 10% and 20% of ethanol as co-solvent) and two UAE extracts (with ethanol and ethanol/water 1:1 *v*/*v*) were obtained from each raw material. The providing yield range was between 2.6 to 76.3 mg/g raw material. The extracts were analyzed with High-Performance Liquid Chromatography coupled with Diode Array Detector (HPLC-DAD) and Liquid Chromatography coupled with High-Resolution Mass Spectrometer (LC-HRMS), and the major compounds, were identified. All the extracts were evaluated for their antioxidant and inhibition activity against collagenase, elastase and tyrosinase enzymes. Grapevine by-product extracts found rich in proanthocyanidins and presented the higher inhibition activity. A holistic green experimental methodology is proposed for the obtainment of extracts from significant medicinal plants by-products that provides us with promising results concerning dermo-cosmetic properties, especially for grape seeds extracts.

## 1. Introduction

Cultivated plants were used from ancient times not only for food, but also for pharmacological purposes [1]. Through the years, the increase of population led at the enlargement of the cultivar production and as a result, at the growth of the produced by-products [2]. Four domesticated plants with significant economic value that their exploitation produce high amounts of wastes are *Vitis vinifera* L. (grapevine)*, Foeniculum vulgare* Mill. (fennel)*, Cannabis sativa* L. (hemp) and *Punica granatum* L. (pomegranate) [3,4]. Grapevine is considered as the most known plant for the production of alcoholic beverages, like wines and spirits [5]. During the fruit processing high amounts of solid wastes are produced, mainly seeds [6]. The same results are observed from the production of pomegranate beverages [7]. The food industry consumes huge quantities of pomegranates for the production of juices and other nutritional products, leading at the generation of seeds and peels that are considered also as wastes [7]. One the other hand, fennel is an aromatic plant, popular for its caudex and roots as flavor enhancement in foods, while its seeds have limited applications [8]. Finally, hemp is one of the most famous medical plants, with a plethora of uses in pharmaceutical, fabric and many other fields, producing seeds as secondary material through the different processes [9].

Nowadays, these seeds are exploited mainly for the production of superior quality vegetable seed oils with use in the cosmetic and food industry [10,11,12,13]. During the oil production procedure, high quantities of by-product seed pastes (VSPs) are generated. Though, they could serve as potential sources of health beneficial compounds and extracts [14,15,16] because they are enriched with the polar constituents of vegetable seeds, named biophenols, known for their strong antioxidant properties. Scientific and industrial interest has focused on exploiting these by-products, since they are reusable and low-cost material suitable for further profitable industry applications. Additionally, by-products of agriculture and food industry due to their rich bioactive content can be used for the replacement of synthetic molecules usage, as antioxidant factors, in food and cosmetic field [17]. Furthermore, the treatment of these kind of by-products minimizes the cultivation wastes and decreasing the environmental fingerprint [18].

In parallel, another need in the industry has arisen in order to fit their production processes with eco-friendly criteria and green methodologies [19]. The last years, the sector of cosmetics adopts the use of non-toxic solvents and materials for the environment and human [20]. At the same time animal tests from research and development methodologies are relinquished and in vivo experiments are switching to in vitro assays [21].

Until today, several green methodologies have been proposed for the treatment of raw materials like VSPs. Specifically, Ultrasound Assisted Extraction (UAE) is a very useful technique to obtain extracts from natural sources [22] UAE by producing cavitation bubbles, increases the shear forces and the solvent is diffused through the walls of the cells [23]. By this way it provokes their collapse, leading to the dispersion of their compounds in the extraction solvent [24]. This methodology allows the use of green solvents, like ethanol (EtOH) and water (H_2_O), leading on the production of edible extracts and products [25].

Likewise, Supercritical Fluid Extraction (SFE) is an entirely green procedure, applicable from lab to industrial scale, with noteworthy advantages. The most common solvent of SFE procedures is carbon dioxide (CO_2_), a non-toxic solvent which is gas at room temperature, so it does not contaminate the final extract [26]. The gaseous nature of CO_2_ is altered on supercritical conditions when the pressure and temperature are adjusted over 72.9 atm and 31.3 °C, respectively [27]. The supercritical form of CO_2_ increases its diffusion properties [28] and effectively penetrates the extraction material. As a result, the mass transfer is increased and the time of extraction is reduced [29]. Another advantage of SFE is that CO_2_, which is considered as a non-polar solvent, can be combined with other more polar green solvents, like EtOH, in order to enrich the extracts with higher polarity molecules [30]. The SFE finds application in a huge range of fields and it has been established as suitable technique for waste and by-product management [31].

Among these lines, in the current study, we aimed to exploit four different by-product pastes, obtained from the cold-pressed oil production processes of grape seeds (*Vitis vinifera*), pomegranate seeds (*Punica granatum*), hemp seeds (*Cannabis sativa*), and fennel seeds (*Foeniculum vulgare*). Two green extraction techniques (UAE and SFE) were employed and two different extracts were produced from each extraction process resulting totally in 16 VSPs extracts. The chemical composition of the produced extracts was investigated using Liquid Chromatography coupled with High-Resolution Mass Spectrometer (LC-HRMS) and High-Performance Liquid Chromatography coupled with Diode Array Detector (HPLC-DAD) analysis.

Thereafter, we aimed to verify that the produced extracts have significant biological activities. Several in vitro assays were performed aiming at the evaluation of their antioxidant activity (Total Phenolic Concentration -TPC and 2,2-Diphenyl-1- picrylhydrazyl -DPPH assays) and for their ability to be used as dermo-cosmetics.

The exposure to ultraviolet radiation results in the production of reactive oxygen species (ROS) which cause cell death if the detoxification is insufficient; therefore, the intake of antioxidants is essential to combat with the oxidative stress [32]. Tyrosinase enzyme is involved in melanogenesis and the overproduction of melanin is responsible for hyperpigmentation skin disorders. Therefore, the pursuit of natural products and agents that inhibit the tyrosinase activity has gained popularity in the cosmetic industry [33]. Another major field of cosmetic research is the skin aging. There have been described two main components of the extracellular matrix that participate in the wrinkle formation, namely elastin and collagen. These proteins provide the skin with elasticity and strength and thus they prevent the aging of the skin. Elastase and collagenase enzymes breakdown these important proteins and therefore several inhibitors have been developed as anti-aging ingredients [34]. In our research study, we performed tyrosinase and collagenase enzymatic activity assay to investigate the anti-aging potential of the produced extracts.

## 2. Results and Discussion

### 2.1. Cold Pressed Production of Seed Oils and By-Product Seed Pastes

All seeds were cold pressed on temperatures between 25 and 30 °C in order to produce oil fractions and their corresponding VSPs by-product. Generally, oil production varies for each kind of seed, depending on the concentration of their saponified content. The highest oil yield was obtained from Hemp (Hmp) seeds (19% *v*/*w* of raw material), followed by Pomegranate (Pmg) seeds and Grape (Grp) seeds (7% *v*/*w* each), while Fennel (Fnn) seeds had the lowest oil production (1.5% *v*/*w*) (Supplementary Table S1). The remaining treated seed pastes (process by-products) constitute the main part of the seeds’ mass (Supplementary Table S1) and are recovered as pellets during the oil production procedure. The duration of oil extraction process varied for each kind of seed, due to the differences on raw material hardness. Despite that the specification of used mill enabled a treatment seed capacity up to 40 kg/h, the operation parameters were set in lower speed in order to avoid thermal degradation of the produced oils (VSOs) and by-product pastes (VSPs).

### 2.2. Recovery of Phenolic Fraction from By-Product Seed Pastes Using SFE and UAE

During the cold pressed VSOs production, certain amounts of paste seeds (VSPs) were produced. The following extraction processes were focused on green criteria in order to produce high quality edible extracts. Two green techniques, SFE and UAE, have been applied leading to the recovery of two fatty fractions and four phenolic extracts of different polarities for each paste material. Table 1 summarize the solvents and extracts’ yields of all obtained fractions.

SFE is a widespread technique in food industry [35], and was applied on seed pastes, in order to obtain two kind of extracts containing mainly the unpolar and middle-polarity constituents. Firstly, a defatting step was necessary to remove the remaining oil from the VSPs. As it is observed from Table 1, all the pastes have a certain amount of fatty fraction. FnnSP provided the highest (123.8 mg/g paste) followed in descending order by PmgSP (89.5 mg/g paste), HmpSP (77.8 mg/g paste) and GrpSP (44.1 mg/g paste). The defatted seed pastes were then extracted with CO_2_ and 10% *w*/*w* of EtOH, as co-solvent. On these conditions, 19.5, 19.0, 2.6 and 31.1 mg/g pastes were obtained from GrpSP, PmgSP, HmpSP and FnnSP, respectively.

The last extraction was conducted with CO_2_ and 20% *w*/*w* of EtOH. The richest extract was produced from FnnSP (49.1 mg/g paste), followed by PmgSP (14.2 mg/g paste), GrpSP (10.0 mg/g paste) and HmpSP (3.9 mg/g paste).

Firstly, 10 g of each different VSPs were defatted with food grade *n-*Hex, providing a lipophilic fraction of 37.8 mg/g paste for GrpSP, 70.6 mg/g paste for PmgSP, 67.5 mg/g paste for HmpSP and 112.6 mg/g paste for FnnSP. Furthermore, ethanolic extracts of VSPs were prepared, providing 55.1 mg/g from GrpSP, 22.7 mg/g from PmgSP, 20.3 mg/g from HmpSP and 34.7 mg/g from FnnSP. The last UAE extracts of VSPs were produced using as extraction solvent H_2_O/EtOH in ratio 1:1 *v*/*v*. Their corresponding yields were 45.6 mg/g, 24.0 mg/g, 23.1 mg/g and 76.3 mg/g for GrpSP, PmgSP, HmpSP and FnnSP, respectively (Table 1).

Taking all the above into consideration, it seems that the defatting process was highly effective for both techniques and for all the raw materials. HPLC data were investigated and they are in accordance with the yield of each extraction (Supplementary Figure S1a–d). Characteristic example is the chromatograms of FnnS extracts, where the CO_2_ 20% EtOH is richer in metabolites (more eluted peaks, with high absorbance) in comparison to CO_2_ 10% EtOH extract (Supplementary Figure S1d). Regarding the hemp seed paste by-products, the SFE-CO_2_ 20% EtOH extraction led to the richest extract in secondary metabolites, showing that this raw material contains mainly unpolar and middle polarity compounds (Supplementary Figure S1a). Furthermore, HPLC peak shaping and baseline, of GrpS extracts showed the existence of polymeric compounds (Supplementary Figure S1a), probably proanthocyanins that have been already identified in the composition of GrpS [36].

### 2.3. LC-HRMS Analysis

For the LC-HRMS analysis, 200 μg/mL of all the extracts were prepared and analyzed by UPLC-ESI-HRMS/MS. The generated chromatograms were studied in parallel with the corresponding spectra and HRMS/MS results. For the identification of compounds, extraction ion method (XIC), peak-to-peak selection, elemental composition (EC) tool, ring and double bonds equivalence (RDBeq) and isotopic patterns were incorporated. For the analyzed extracts, all the detected *m*/*z* values were registered in combination with the above-mentioned information. In parallel, a comparative quantitative analysis based on the peak area of each identified compound, using XIC, was performed in all the extracts. The aim of this analysis was the investigation of the extracts’ chemical composition, as well as to evaluate the efficiency of the different extraction techniques concerning the recovery of each compound.

The LC-HRMS analysis showed that all the analyzed extracts contain mainly organic acids and phenolic compounds. Furthermore, each raw material contained different categories of phenolic compounds (Supplementary Figure S2a–d). Table 2 summarizes all the identified organic acids and phenolic compounds of grape, pomegranate, hemp and fennel by-product seed extracts. Besides, Table 2 reveals the relative quantity of identified molecules in the four different extracts obtained from SFE and UAE techniques.

The LC-HRMS analysis of the extracts obtained from grape seed paste by-product led to the tentative identification of 25 secondary metabolytes of which three were organic acids, two were phenolic acid derivatives, three were flavanols and their derivatives and 17 were proanthocyanidins (Table 2). Catechin and epicatechin were the major phenolic compounds of ethanolic and hydroalcoholic extract obtained from UAE extraction, while their recovery by SFE is propotional to the percentage of EtOH used as co-solvent. Proanthocyanidins is an important class of bioactive compounds, common in grape skin and seeds [56]. Based on the above analysis, SFE extraction, with CO_2_ and 10% EtOH as co-solvent, is not capable to obtain proanthocyanidins from grape seed paste. On the other hand, SFE extraction with CO_2_ and 20% EtOH led to the partial recovery of dimer proanthocyanidin derivatives. Moreover, the most polar solvent systems (EtOH and EtOH/H_2_O) used in UAE extractions, led to the efficient recovery of not only dimer, but also trimer and tetramer proanthocyanidin derivatives (Table 2).

Fourteen metabolites were tentatively identified by LC-HRMS analysis of hemp seed paste extracts (Table 2). From those, seven were canabinoid acid derivatives, four lignamides (Cannabisin A, B and C), two amides (trans and cis caffeoyltyramine) and one phenolic acid. It is important to note that the extraction of raw material by SFE technique and the solvent system CO_2_ + 10% of EtOH resulted in better recovery of cannabinoid acid derivatives, while the same extraction method with the addition of 20% of EtOH as co-solvent led to more effective recovery of alcaloid compounds (amides and lignamides).

The analysis of pomegranate seed paste extracts revealed a poor phenolic profile of these extracts which led to the identification of 10 constituents, four belonging to phenolic acids and only one to flavonoids (Table 2). The same analysis of fennel seed paste extracts led to the tentative identification of 11 secondary metabolites of which four were flavonoid derivatives and two caffeic acid derivatives (Table 2).

Overall, the extraction pipeline was efficient to provide sixteen extracts with different secondary metabolites. Due to the fact that these metabolites have been previously described in the literature, we have have not performed factorial design for the isolation of the compounds and this stands as a limitation of our study.

### 2.4. Biological Evaluation of VSO and VSP Extracts

#### 2.4.1. TPC and DPPH of Extracts

Both DPPH and TPC are two established methodologies for the evaluation of the antioxidant capacity of extracts and/or pure compounds, widely used in the field of natural products [57,58].

TPC is a methodology for the determination of polyphenols. This technique measures the total reducing capacity of a sample and results are expressed as mg of gallic acid equivalents (GAE) per g of extracts [57]. The extracts were tested in two concentrations, 500 and 1000 μg/mL and our results demonstrated the proper correlation between the estimated values in all cases. The estimated mean values showed a considerable variation 16.07–324.84 mg GAE/g. In more detail, the highest values were recorded for GrpS. More specifically, UAE EtOH/H_2_O presented the highest value (324.84 mg GAE/g) followed by UAE EtOH (253.19 mg GAE/g) and SFE 20% (147.26 mg GAE/g). Values of GrpS extracts are the highest of the dataset, indicating the high phenolic content of GrpS in comparison to the other tested raw materials. It has to be noted that from the four tested seed extracts, two UAE extracts revealed the highest TPC values, followed by the one SFE extract with 20% of EtOH as co-solvent, underlining the efficiency of both extraction methodologies for the obtainment of phenolic compounds (Supplementary Figure S3).

DPPH is considered as an accurate methodology for the evaluation of the radical scavenging activity of antioxidants and it is expressed as percentage (%) of oxidative inhibition [58]. In our results, four different concentrations (500, 250, 50 and 25 μg/mL) of each extract were tested reaching an inhibition range of −3.06% to 63.5%. As it was expected from TPC assays, the most effective radical scavenging activity was found in GrpSP extracts. In specific, 500 μg of UAE EtOH/H_2_O 1:1 *v*/*v* extract showed 63.5 ± 5.2% inhibition, followed by 55.9 ± 5.6%, 47.9 ± 1.2% and 31.2 ± 1.5% for the 250, 50 and 25 μg/mL, respectively. Furthermore, the GrpSP extracts of UAE EtOH and SFE 20% EtOH exhibited significant inhibition. The highest concentration of HmpSP SFE 20% EtOH extract gave positive results as well (Supplementary Figure S4). The high antioxidant activity of these extracts was in accordance with LC-HRMS data that demonstrated their chemical profiles, which were rich in phenolic molecules.

Interestingly, GrpSP has similar and even higher antioxidant capacity compared to extracts from other plant parts or extracts that have been produced with other methodologies [4]. Notably, the phytochemical content of the grape seed extracts is rich in antioxidant compounds such as gallic acid, proanthocyanidins [59,60]. The antioxidant capacity of hemp UAE extracts provides higher level than other scientific research due to the different solvent extraction [61]. Pomegranate and fennel seed pastes is show lower antioxidant activity than other by-product pastes which include different parts of the same plant [62,63,64]. However, the important finding of the study is that the antioxidant activity of GrSP is similar to the one reported for seeds [65].

#### 2.4.2. Enzymatic Inhibition Activity of Extracts

Tyrosinase, collagenase and elastase enzymes are involved in the pigmentation and aging possesses as discussed in detail in the introduction. Thus, the activity assays for these enzymes are used to test the efficiency of extracts and pure compounds for dermo-cosmetic applications [33,66,67]. All the above-mentioned extracts from VSPs were evaluated for their inhibition properties on these three enzymes.

Pomegranate fruit concentrated solutions and peels are well-studied products of *Punica granatum* and many studies have evaluated their potent inhibition skill against collagenase, elastase and tyrosinase [68,69,70]. However, there are no reports available for the inhibitory activity, against these enzymes, of PmgSP extracts. Additionally, fennel fruits, they have been found as inactive inhibitors for elastase and tyrosinase enzymes in previous studies [71,72]. Moreover, cannabis leaves provide low tyrosinase inhibition activity while seeds have no significant activity against this enzyme [73,74]. Several studies have proven that grape seed and pomace have anti-collagenase, anti-elastase and anti-tyrosinase properties due to their high proanthocyanidins content [75,76,77,78]. Based on the above literature data, information regarding enzymatic inhibition studies of the four VSPs are scarce. Towards this purpose, all the investigated materials were evaluated concerning their inhibitory properties against tyrosinase, collagenase and elastase enzymes.

**Figure 1:**
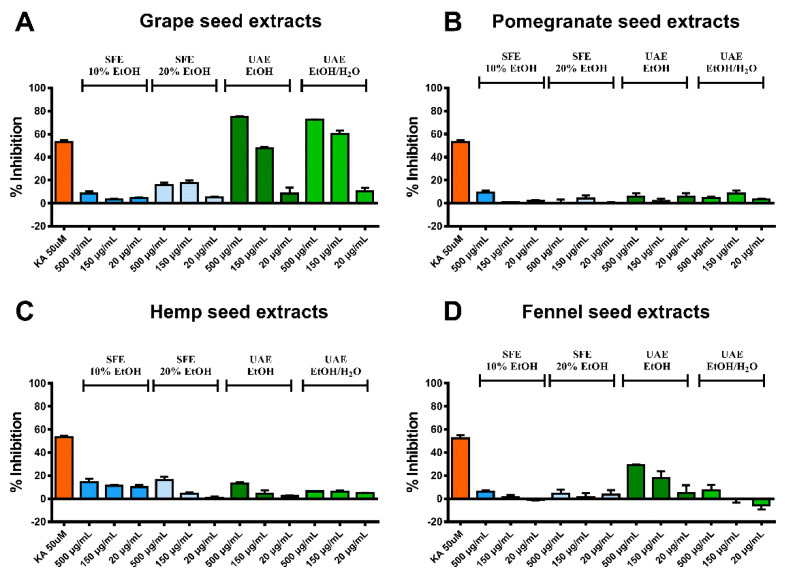
Results of the tyrosinase activity assay. The percentage of tyrosinase enzyme inhibition (%) is presented for the produced extracts from (**A**) grape seeds, (**B**) pomegranate seeds, (**C**) hemp seed and (**D**) fennel seeds. Kojic acid (KA) was used as positive control.

Starting with tyrosinase activity assay, only the two UAE extracts (EtOH and EtOH/H_2_O 1:1 *v*/*v*) of GrpSP revealed significant inhibition on tyrosinase enzyme. Specifically, the concentration of 500 μg/mL provided 75.0 ± 0.7% and 72.4 ± 0.3% inhibitory activity, while 150 μg/mL of the UAE EtOH and UAE EtOH/H_2_O extracts showed 48.0 ± 1.0% and 60.4 ± 2.7% inhibition, respectively, suggesting the anti-pigmentation properties of GrpS (Figure 1A). All the other tested pastes showed inhibition activities below the positive control (kojic acid), even at the higher concentrations (% of inhibition for the positive control: 52.8 ± 2.0%) (Figure 1B–D).

**Figure 2:**
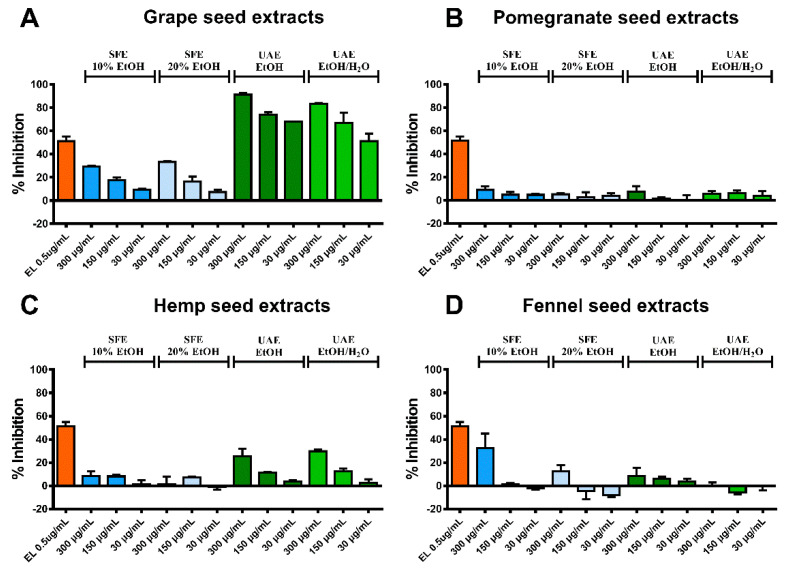
Results of the elastase activity assay. The percentage of elastase enzyme inhibition (%) is presented for the produced extracts from (**A**) grape seeds, (**B**) pomegranate seeds, (**C**) hemp seed and (**D**) fennel seeds. Elastatinal (EL) was used as positive control.

Regarding the elastase activity assay, the two GrpSP UAE extracts (EtOH and EtOH/H_2_O 1:1 *v*/*v*) exhibited remarkable high inhibitory activity on the elastase enzyme at all tested concentrations (30, 150 and 300 μg/mL). Specifically, GrpSP UAE EtOH at 30, 150 and 300 μg/mL showed inhibition of 67.8 ± 0.5%, 74.0 ± 2.1% and 91.3 ± 1.4%, respectively, and GrpSP UAE EtOH/H_2_O 51.3 ± 6.4%, 62.8 ± 8.2% and 83.2 ± 1.0%, at the respective concentrations. (Figure 2A). The positive control of the assay, Elastatinal (IC_50_ = 0.5 µg/mL), exhibited percentage of inhibition 51.3 ± 3.7%. Interpreting the results of this assay, it is evident that GrpS extracts strongly inhibit elastase enzyme and can be suggested as an anti-aging agent.

**Figure 3:**
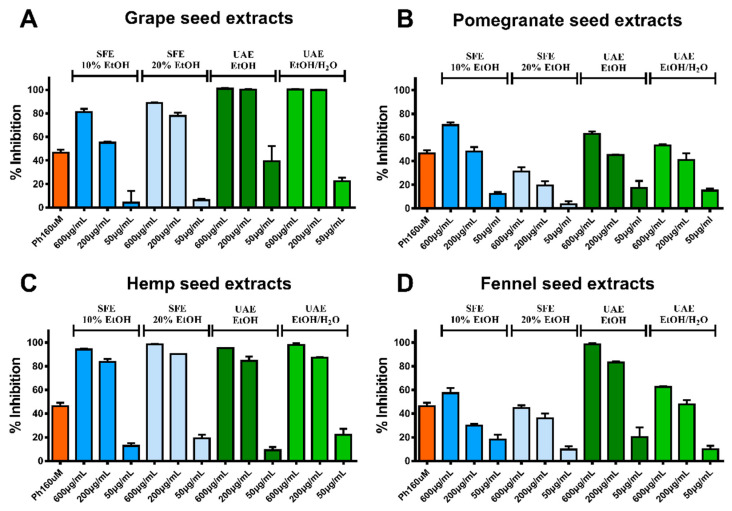
Results of the collagenase activity assay. The percentage of collagenase enzyme inhibition (%) is presented for the produced extracts from (**A**) grape seeds, (**B**) pomegranate seeds, (**C**) hemp seed and (**D**) fennel seeds. Phosphoramidon (PRMD) was used as positive control.

In respect of collagenase activity assay, numerous of the tested extracts showed inhibitory activity. As it was expected for GrpSP all the tested extracts revealed inhibitory activity, with the UAE extracts (EtOH and EtOH/H_2_O 1:1 *v*/*v*) reaching approximately 100% inhibition (Figure 3A). Additionally, all HmpS extracts revealed inhibitory activity over 80% (600 μg/mL of UAE EtOH, UAE EtOH/H_2_O, SFE 10% and SFE 20% for 95.2 ± 0.3%, 98.0% ± 1.3, 94.2 ± 0.5% and 98.5 ± 0.2%, respectively), as well as FnnSP UAE EtOH (98.3 ± 1.0% at 600 μg/mL). Significant inhibition was also evident for FnnSP UAE EtOH/H_2_O (62.4 ± 0.8% at 600 μg/mL), FnnSP SFE10% (57.3 ± 4.3% at 600 μg/mL), PmgSP UAE EtOH (63.0 ± 1.9% at 600 μg/mL) and PmgSP SFE10% (70.3 ± 2.3% at 600 μg/mL) showed inhibition activity over IC_50_ of the positive control phosphoramidon (IC_50_ = 16 µM, percentage of inhibition for collagenase enzyme 46.4 ± 2.8%) (Figure 3B–D).

It has to be emphasized that GrpSP extracts revealed significant inhibition activity against all enzymes, indicating their high efficiency as potential starting materials for the manufacture of dermo-cosmetic products.

Taking into account, the metabolites that are present in each extract and their biological activity several conclusions may be drawn. As it is already mentioned, Grp seeds were the only raw material, which extracts showed inhibition activity against all enzymes. Studying Table 2 and in parallel with bibliographic investigation, it can be assumed that GrpSP extracts are rich in proanthocyanidins like dimer of epicatechin/catechin, dimer proanthocyanidin galloylated and trimer proanthocyanidin. Based on literature data these metabolites are characterized by anti-elastase, anti-collagenase and anti- tyrosinase activity [79,80,81]. The higher amount of the above proanthocyanidins derivatives in UAE extracts compare to SFE extracts (Table 2) could explain the different inhibition activity of these extracts against tyrosinase, elastase and collagenase. Hmp seed extracts showed inhibition activity against collagenase enzyme. Based on Table 2, the extract was rich in CBD A, cannabisin A, B and C, compounds that seem to enhance the anti-collagenase activity. It has to be noted that these compounds despite being followed by a long bibliographic research for their biological activities and their promising results for human health [82,83] they have never been investigated before for their tyrosinase, elastase and collagenase activity. Moreover, Fnn seeds showed anti-collagenase activity. In these extracts, casearicoside A, corchorifatty acid, dihydroxy-octadecenoic acid and myristicin were identified and the potent activity of the extracts could be attributed to some of these molecules and/or synergistic effect of these compounds. PmgSP SFE 10% was mostly enriched in phenolic acids (gallic acid, quinic acid, protocatechuic acid, 4-hydroxybenzoic acid, vanillic and coumaric acid) and kaempherol, agents that could account for its inhibitory effect on collagenase enzyme. Lastly, SnfSP UAE EtOH/H_2_O and PmgSP SFE 10% were characterized by phenolic acids (chlorogenic acid, coumaroylquinic acid, 3-O-feruloylquinic acid, caffeic acid, hydroxycaffeic acid, Di-O-caffeoylquinic acid, isoferulic acid).

## 3. Materials and Methods

### 3.1. Reagents and Materials

The extraction solvents were of analytical grade and purchased from Thermo Fisher Scientific (Waltham, MA, USA). The solvents used in UPLC-HRMS analysis were LC-MS grade and supplied also from Thermo Fisher Scientific (Waltham, MA, USA). Gallic acid, sodium carbonate (99% purity), 2,2-diphenyl-1-picrylhydrazyl (DPPH, 95% purity), Folin–Ciocalteu reagent and the reagents of the enzymatic assays were purchased from Sigma-Aldrich. In detail, mushroom tyrosinase (lyophilized powder, ≥1000 units/mg solid, EC Number: 1.14.18.1), 3,4-dihydroxy-L-phenylalanine, sodium phosphate monobasic, sodium phosphate dibasic, kojic acid, elastase type IV from porcine pancreas (EC Number 254-453-6), N-Succinyl-Ala-Ala-Ala-p-nitroanilide (EC Number 257-823-5), Trizma base reagent grade, elastatinal, collagenase from Clostridium histolyticum (released from physiologically active rat pancreatic islets Type V, ≥1 FALGPA units/mg solid, >125 CDU/mg solid (EC Number: 232-582-9), MMP 2 fluorogenic substrate (MCAPro-Leu-Ala-Nva-DNP-Dap-Ala-Arg-NH_2_) and phosphoramidon were purchased also from Sigma-Aldrich.

The raw material was provided from Greek farmers. *Vitis vinifera* seeds, a by-product material of the wine industry, was provided from farmers of Messinia area. The seeds of *Foeniculum vulgare* were obtained from producers of Attiki area, *Cannabis sativa* seeds obtained from industrial hemp variety Fedora, cultivated at Thessalian valey, while *Punica granatum* seeds were collected from Argolida.

### 3.2. Cold Press Production of Seed Oils and By-Product Seed Paste

The seeds (Supplementary Table S1) were cold pressed using a KK40 F Special (Oil Press GMPH, Reut, Germany) food-safe mill. The seeds are placed in the stainless-steel hopper and passed through a system of hard seed screws and sieves where the material is pressed producing the seed oil and pellets of oil-free paste. The specification of mill enabled a seed capacity of up to 40 kg/h, depending on seed morphological characteristics (seed size, hardness, oil percentage, etc.). In the present work, the screws speed was properly adjusted to cover treatment capacity of 15 kg/h for the hardest seeds (*GrpS* and *PmgS*), and 25 kg/h for the softer seeds (*HmpS* and *FnnS*). Table S1 in supplementary summarizes the procedure parameters followed for the cold press production of seed oils (VSOs) and remaining seed pastes (VSPs).

### 3.3. Production of Green Extracts Using UAE and SFE

The generated VSPs were extracted with two different methods, UAE and SFE. Ultrasound extractions took place in an ultrasonic P300H bath of Elma Schmidbauer (Singen, Germany). 10 g of each VSP were placed in erlenmeyers and extracted successively with 30 mL of *n-*Hex, EtOH and EtOH/H_2_O 1:1 (*v*/*v*). Each ultrasound extraction lasted 20 min and the corresponding eluents were evaporated to dryness and weighted. For the supercritical fluid experiments, analytical scale apparatus of SEPAREX (Nancy, France) was used. It was consisted of a force ventilation oven, which was equipped with a 100 mL stainless steel extraction vessel, a separator, a back-pressure regulation valve, a CO_2_ chiller unit, a co-solvent and a CO_2_ liquid pump. The sample vessel has a total volume of 100 mL and 50 g from each paste were initially extracted with 100% CO_2_ a flow rate of 15 g/min. After the defatting step, two more successive extractions were applied. The first one was accomplished with CO_2_ at 15 g/min and 10% EtOH *w*/*w* as co-solvent and the second one with 15 g/min CO_2_ and 20% EtOH *w*/*w*. In all the SFE procedures, pressure was set at 300 bar and extracts were treated until exhaustiveness.

### 3.4. HPLC-DAD and LC-HRMS Analysis of the Extracts

HPLC analysis was conducted on a Thermo Finnigan HPLC system (Ontario, Canada) equipped with a SpectraSystem P4000 pump, a SpectraSystem 1000 degasser, a SpectraSystem AS3000 automated injector, and a UV SpectraSystem UV6000LP detector. Data acquisition was controlled by the ChromQuest™ 5.0 software (ThermoScientific™). HPLC-DAD experiments were run on a Supelco Analytical (Sigma-Aldrich) HS C18 column, with dimensions 25 cm × 4.6 mm, 5 μm, at room temperature. As elution solvent system, acidified H_2_O (0.1% *v*/*v* of formic acid) and acetonitrile were used. Gradient was started with 98% of H_2_O and reached 2% after 60 min. At 61^st^ min, the elution system returned to the initial conditions and stayed for 4 min. Flow rate was set at 1 mL/min and injection volume at 10 μL. Chromatograms of 254 nm, 280 nm and 366 nm, were recorded.

All the produced extracts were analyzed with UPLC-HRMS/MS. The experiments were accomplished on an H class Acquity UPLC system (Waters, Milford, CT, USA) coupled to an LTQ-Orbitrap XL hybrid mass spectrometer (Thermo Fisher Scientific, Waltham, CT, USA). For the chromatographic separation, a Fortis C-18 column (1.7 µm, 150 × 2.1 mm) was used, and temperature was set at 40 °C. The elution solvent system was consisted of acidified water with 0.1% formic acid (A) and acetonitrile (B). Gradient started with 2% B for 2 min, which reached 100% at 18 min and stayed for 2 more min. At the 21st minute, system returned to the initial conditions and stayed for 4 min for system equilibration. The flow rate was at 400 μL/min, the injection volume was 10 µL and samples were kept at 7 °C. Ionization was carried out in negative and positive ion mode (ESI±). The mass spectrometric parameters were: capillary temperature 350 °C; sheath gas 40 units; aux gas 10 units; capillary voltage 30 V; and tube lens 100 V for the positive mode. For negative ionization capillary voltage was adjusted at −20 V and tube lens of −80 V, while all the other parameters remained stable. Data were recorded in full scan from 113 to 1000 *m*/*z* and HRMS/MS experiments were carried out with data dependent method with collision energy 35.0% (q = 0.25).

### 3.5. TPC and DPPH Assays

Total Phenolic Content (TPC) of extracts was evaluated via Folin Ciocalteu colorimetric assay. Gallic acid was used for TPC evaluation and eight different concentrations were applied for the construction of the calibration curves (2.5 μg/mL, 5 μg/mL, 10 μg/mL, 12.5, 20, 25, 40 and 50 μg/mL) mixed with Folin–Ciocalteu reagent (ten-fold dilution) and sodium carbonate solution (7.5% *w*/*v*). Extracts were tested in two different concentrations (1000 and 500 μg/mL in the wells) and analyzed twice in triplicates. The absorptions were measured on an Infinite 200 PRO series reader (Tecan Group, Männedorf, Switzerland) at 765 nm. TPC values were expressed as mg of gallic acid equivalent/mg of extract using the resulted calibration curve (R^2^ = 0.9978).

DPPH radical scavenging assay was performed using a previously described protocol [84] with some modifications. DPPH stock solution was prepared by diluting 12.4 mg of DPPH reagent in 100 mL absolute ethanol reaching the final concentration of 314 μΜ. Stock solution was vortexed and stored in dark at room temperature until analysis day. As positive control gallic acid was used, in concentration of 29.4 µM. All the extracts were diluted in DMSO providing four different concentrations of 10 mg/mL, 5 mg/mL, 1 mg/mL and 0.5 mg/mL. Briefly, 190 μL of DPPH solution were mixed with 10 μL of gallic acid or the samples in a 96-well plate. Negative control was composed of 190 μL of DPPH solution and 10 μL of DMSO. For the blanks 190 μL of EtOH and 10 μL of sample were used. All the experiments were conducted twice in triplicates. After 30 min incubation in dark place and at room temperature, the absorbance (abs) was measured using an Infinite 200 PRO series reader (Tecan Group, Männedorf, Switzerland) at 517 nm. The calculation formula of radical scavenging activity percentage is:

radical scavenging activity (%) = [1 − ((X sample − X blank) / X control)] × 100(1)

X control symbolizes the absorbance of the negative control, while X sample the absorbance after the reaction of samples with DPPH. X blank symbolizes the absorbance of samples with EtOH.

### 3.6. Tyrosinase, Elastase and Collagenase Activity Assays

The enzymatic assays for tyrosinase, elastase and collagenase enzymes were applied as previously described by Michailidis et al. and Angelis et al. [66,67] with a few modifications. Experiments were performed twice in triplicates. The calculation formula of inhibition percentage is:

Inhibition (%) = [((X control − X control’s blank) − (X sample − X sample’s blank)) / (X control − X control’s blank)] × 100(2)

X control symbolizes the absorbance or fluorescence value of the mixture consisted of buffer, enzyme, sample solvent, and substrate, while X sample stands for the absorbance or fluorescence value of the mixture of buffer, enzyme, sample or positive control solution and substrate. Blanks contained all the above-mentioned components except the enzyme.

As standard of comparison for tyrosinase, elastase, and collagenase enzymatic assays the half maximal inhibitory concentration (IC50) of each positive control was used.

Tyrosinase enzymatic assay: This assay evaluates the action of the tested samples at the catalytic oxidation of L-DOPA to dopachrome by tyrosinase. Kojic acid (IC_50_ = 50 µM, percentage of inhibition for tyrosinase enzyme 52.8 ± 2.0%) was used as positive control. In a 96-well microplate, 80 µL of phosphate-buffered saline (PBS) (0.067 M, pH = 6.8), 40 µL of the tested sample, and 40 µL of mushroom tyrosinase (EC Number: 1.14.18.1), 100 U/mL dissolved in PBS buffer were mixed and incubated in the dark for 10 min at room temperature. Afterwards, 40 µL of 2.5 mM L-DOPA (substrate) dissolved in PBS buffer were added and the mixture was incubated for 10 min so that the dopachrome is formed. The tyrosinase activity was determined at 475 nm using the reader Infinite 200 PRO series (Tecan). The final concentrations of the extracts on the plate were 500, 150 and 20 μg/mL diluted in PBS with 1% DMSO.

Elastase enzymatic assay: The evaluation of elastase activity is based on the release of p-nitroaniline from N-succinyl-Ala-Ala-Ala-p-nitroanilide that is stimulated by elastase. Elastatinal (IC_50_ = 0.5 µg/mL, percentage of inhibition for elastase enzyme 51.3 ± 3.7%) was used as positive control. In a 96-well microplate, 70 μL of Tris-HCl buffer (50 mM, pH = 7.5), 10 µL of tested sample, and 5 µL of elastase (0.45 U/mL) (dissolved in Tris-HCl buffer) were mixed and incubated in the dark for 15 min at room temperature. Then, 15 μL of 2 mM N-succinyl-Ala-Ala-Ala-p-nitroanilide (substrate) dissolved in Tris-HCl buffer was added, and the mixture was incubated for 30 min at 37 °C. The production of p-nitroaniline was determined at 405 nm using the reader Infinite 200 PRO series (Tecan). The final concentrations of the extracts were 300, 150 and 30 μg/mL dissolved in Tris-HCl buffer with 0.5% DMSO. Collagenase enzymatic assay: Collagenase activity was determined with a spectrofluorimetric method using a fluorogenic metalloproteinase-2 (MMP2) substrate (MCAPro-Leu-Ala-Nva-DNP-Dap-Ala-Arg-NH_2_) which is enzymatically degraded by collagenase to produce fluorescence. Phosphoramidon (IC_50_ = 16 µM, percentage of inhibition for collagenase enzyme 46.4 ± 2.8%) was used as positive control. In a 96-well black microplate, 120 µL of Tris-HCl buffer (50 mM, pH = 7.3), 40 μL of tested sample, and 40 µL of collagenase (50 µg/mL) from *C. histolyticum* (dissolved in Tris-HCl buffer) were incubated for 10 min at 37 °C avoiding light exposure. Then, 40 µL of 50.0 µM fluorogenic substrate dissolved in Tris-HCl buffer were added, and the mixture was incubated in dark for 30 min at 37 °C. The fluorescence intensity was measured at an excitation maximum of 320 nm and an emission maximum of 405 nm. All the measurements were carried out in the Infinite 200 PRO series reader (Tecan Group, Männedorf, Switzerland).

## 4. Conclusions

The seeds of four plants with high medicinal interest, grapevine, hemp, pomegranate and fennel were used for the production of VSOs and VSPs. Green extracts from VSPs were investigated for their anti-collagenase, anti-elastase and anti-tyrosinase inhibition activity, while in parallel a broad range chemical characterization was conducted. Moreover, all the extracts were analyzed with HPLC-DAD and LC-HRMS. Combination of these techniques provided information about the total chemical composition of the starting raw materials. It should be mentioned that the extraction methodologies used in the present study, UAE and SFE, were based on low environmental fingerprint techniques with non-toxic solvents, providing totally green extracts. All the above-mentioned extracts were applied on enzymatic assays in order to evaluate their dermo-cosmetic properties. Most of them were found to be strong anti-collagenase inhibitors, while UAE grape seeds show significant results and as anti-elastase and anti-tyrosinase factors. Concluding, based on our in vitro data there is a correlation between the antioxidant activity-enzymatic inhibition activity and extracts’ solutes. Our methodology proposes novel methods for the exploitation of oil production wastes by the cosmetic industry while the antioxidant effect of our extracts renders them ideal candidates for nutritional and dermo-cosmetic products. Since the grape seed extracts possessed the greatest biological significance, we will pursue in the future the implementation of our methodology at industry scale and the incorporation of these extracts in commercially available cosmetic products. We believe that green based methodologies will be the cornerstone of the economic utilization of plant by-products.

## Figures and Tables

**Table 1:** Yields of seed paste (VSP) extracts (mg/g of raw material) from grape (GrpSP), pomegranate (PmgSP), cannabis (HmpSP) and fennel (FnnSP) after UAE with *n*-Hex, EtOH and EtOH/H_2_O 1:1 *v*/*v* and SFE with CO_2_, CO_2_ with 10%EtOH *w*/*w* and CO_2_ with 20%EtOH *w*/*w*.

Extraction Solvent	UAE			SFE		
	*n*-Hex Defatting (mg/g Raw Material) (*w/w* Percentage of Raw Material)	EtOH (mg/g Raw Material)	EtOH/H_2_O 1:1 *v/v* (mg/g Raw Material)	CO_2_ Defatting (mg/g Raw Material) (*w/w* Percentage of Raw Material)	CO_2_ + 10%EtOH *w/w* (mg/g Raw Material)	CO_2_ + 20%EtOH *w/w* (mg/g Raw Material)
GrpSP	37.8 (3.8%)	55.1	45.6	44.1 (4.4%)	19.5	10.0
PmgSP	70.6 (7.1%)	22.7	24.0	89.5 (9.0%)	19.0	14.2
HmpSP	67.5 (6.8%)	20.3	23.1	77.8 (7.8%)	2.6	3.9
FnnSP	112.6 (11.3%)	34.7	76.3	123.8 (12.4%)	31.1	49.1

**Table 2:** The list of tentatively identified metabolites in the by-product seed extracts by Ultra Performance Liquid Chromatography equipped with Electron Spray Ionization source coupled with High Resolution Mass Spectrometer UPLC-ESI-HRMS/MS-orbitrap.

Grape Seed Paste By-Product										
Proposed Compound	Rt (min)	EC	*m*/*z* exp.	Delta (ppm)	RDB	Ref.	Extract (Peak Area × 10^6^)			
							SFE-CO_2_		UAE	
							10% EtOH	20% EtOH	EtOH	EtOH/H_2_O
**Gluconic acid**	0.59	C_6_H_12_O_7_	195.0515	2.73	1.5	[37]	4.25	1.98	3.19	1.83
**Tartaric acid**	0.71	C_4_H_6_O_6_	149.0097	3.62	2.5	[37]	2.21	13.1	1.37	0.91
**Citric acid**	0.91	C_6_H_8_O_7_	191.0202	2.59	3.5	[37]	1.19	12.2	1.58	1.67
**Gallic acid**	1.35	C_7_H_6_O_5_	169.0147	2.62	5.5	[37]	18.0	18.1	5.96	1.77
**Glucogallin**	2.84	C_13_H_16_O_10_	331.0673	0.57	6.5	[38]	3.07	19.5	4.76	1.08
**Epicat./cat.**	4.96	C_15_H_14_O_6_	289.0723	1.97	9.5	[39]	0.04	40.3	32.7	48.2
**Epicat./cat.**	5.35	C_15_H_14_O_6_	289.0722	1.41	9.5	[39]	0.09	64.1	43.4	61.2
**Epicat. galate**	6.33	C_22_H_18_O_10_	441.0832	1.07	14.5	[39]	-	16.1	5.58	9.27
**Dimer proanth**	4.79	C_30_H_26_O_12_	577.1348	0.51	18.5	[40]	-	10.6	15.1	23.6
	5.20	C_30_H_26_O_12_	577.1361	1.67	18.5	[40]	-	12.2	13.4	20.1
	5.30	C_30_H_26_O_12_	577.1360	1.47	18.5	[40]	-	8.78	35.6	52.3
**Dimer proanth gall**	5.58	C_37_H_30_O_16_	729.1470	1.18	23.5	[41]		1.51	1.04	1.67
	5.80	C_37_H_30_O_16_	729.1468	0.94	23.5	[41]	-	7.51	18.8	27.3
**Trimer proanth**	4.92	C_45_H_38_O_18_	865.1979	−0.56	27.5	[42]	-	0.36	1.89	2.74
	5.07	C_45_H_38_O_18_	865.1986	0.09	27.5	[42]	-	0.72	2.01	2.81
	5.37	C_45_H_38_O_18_	865.1985	0.36	27.5	[42]	-	1.52	3.12	4.45
	5.67	C_45_H_38_O_18_	865.1984	−0,12	27.5	[42]	-	2.41	5.26	7.56
	6.14	C_45_H_38_O_18_	865.1989	0.50	27.5	[42]	-	0.61	5.67	7.64
**Trimer proanth.-gall**	5.37	C_52_H_47_O_22_	1017.2103	0.76	32.5	[42]	-	-	1.35	2.27
	5.77	C_52_H_47_O_22_	1017.2093	−0.20	32.5	[42]	-	-	1.53	2.46
	6.30	C_52_H_47_O_22_	1017.2098	0.28	32.5	[42]	-	-	1.97	3.44
**Tetramer proanth.**	5.11	C_60_H_49_O_24_	1153.2622	0.25	36.5	[43]	-	-	0.48	1.05
	5.61	C_60_H_49_O_24_	1153.2630	0.98	36.5	[43]	-	-	0.69	1.17
	5.75	C_60_H_49_O_24_	1153.2621	0.13	36.5	[43]	-	-	1.36	2.36
	6.01	C_60_H_49_O_24_	1153.2613	−0.50	36.5	[43]	-	-	0.66	1.17
**Pomegranate Seed Paste By-Product**										
**Proposed Compound**	**Rt (min)**	**EC**	***m*** **/*z* exp.**	**Delta** **(ppm)**	**RDB**	**Ref.**	**Extract (Peak Area × 10^6^)**			
							**SFE-CO_2_**		**UAE**	
							**10% EtOH**	**20% EtOH**	**EtOH**	**EtOH/H_2_O**
**Gulonic acid**	0.60	C_6_H_11_O_7_	195.0514	1.713	1.5	[44]	19.9	45.9	27.9	24.7
**Glucopyranose**	0.67	C_6_H_11_O_6_	179.0565	2.171	1.5	[45]	29.2	31.3	18.2	16.8
**Gallic acid**	1.37	C_7_H_5_O_5_	169.0149	3.748	5.5	[44]	1.84	3.97	2.36	1.38
**Quinic acid**	1.42	C_7_H_11_O_6_	191.0566	2.505	2.5	[44]	2.01	-	0.33	-
**Protocatechuic acid**	2.73	C_7_H_5_O_4_	153.0199	3.712	5.5	[45]	3.31	2.04	1.63	0.86
**4-Hydroxybenzoic acid**	4.30	C_7_H_5_O_3_	137.0251	4.753	5.5	[45]	5.48	0.04	1.53	0.43
**Vanillic acid**	5.01	C_8_H_7_O_4_	167.03552	3.221	5.5	[44]	1.14	0.07	0.38	0.13
**Couma** **ric acid**	5.91	C_9_H_7_O_3_	163.04056	3.021	6.5	[46]	0.75	0.10	0.11	0.14
**Kaempferol**	8.60	C_15_H_9_O_6_	285.04089	1.504	11.5	[46]	0.33	0.23	0.09	0.04
**Asiatic acid**	11.08	C_30_H_47_O_5_	487.34357	1.379	7.5	[46]	2.06	-	1.49	0.50
**Hemp Seed Paste By-Product**										
**Proposed Compound**	**Rt (min)**	**EC**	***m*** **/*z* exp.**	**Delta** **(ppm)**	**RDB**	**Ref.**	**Extract (Peak Area × 10^6^)**			
							**SFE-CO_2_**		**UAE**	
							**10% EtOH**	**20% EtOH**	**EtOH**	**EtOH/H_2_O**
**Hydroxybenzoic acid**	7.05	C_7_H_5_O_3_	137.0251	4.613	5.5	[47]	80.9	70.1	2.42	12.4
**Cannabinolic acid**	12.93	C_22_H_25_O_4_	353.1764	1.578	10.5	[48]	3.99	0.60	0.32	0.28
**Cannabichromevarinic acid**	13,31	C_20_H_25_O_4_	329.1762	1.056	8.5	[47]	5.81	0.25	0.83	0.63
**Caffeoyl tyramine** **isomer I**	6.65	C_17_H_16_O_4_N	298.1086	0.633	10.5	[47]	16.5	122.3	7.62	13.7
**Caffeoyl tyramine** **isomer II**	6.91	C_17_H_16_O_4_N	298.1084	−0.273	10.5	[47]	20.9	112.1	11.5	26.3
**Cannabisin A**	7.40	C_34_H_29_O_8_N_2_	593.1927	0.319	21.5	[49]	-	9.50	3.97	5.54
**Cannabisin B** **isomer I**	7.43	C_34_H_31_O_8_N_2_	595.2086	0.049	20.5	[50]	0.37	42.1	11.2	22.1
**Cannabisin B** **isomer II**	8.76	C_34_H_31_O_8_N_2_	595.2089	0.657	20.5	[50]	0.49	28.5	3.74	5.69
**Cannabisin C**	7.85	C_35_H_33_O_8_N_2_	609.2243	0.100	20.5	[50]	1.24	57.5	3.05	9.65
**Cannabidiolic acid**	14.34	C_22_H_29_O_4_	357.2073	0.496	8.5	[48]	93.2	7.16	27.2	13.2
**(±)-6,7-cis/trans-epoxy cannabigerolic acid**	12.41	C_22_H_31_O_5_	375.2164	−0.454	7.5	[51]	0.76	0.08	0.09	0.20
**α** **/** **β** **-cannabielsoic acid** **isomer I**	11.29	C_22_H_29_O_5_	373.2022	0.516	8.5	[51]	10.4	1.73	0.93	0.67
**α** **/** **β** **-cannabielsoic acid** **isomer II**	12.47	C_22_H_29_O_5_	373.2023	0.758	8.5	[51]	8.43	1.37	0.51	0.62
**α** **/** **β** **-cannabielsoic acid** **isomer III**	13.60	C_22_H_29_O_5_	373.2027	0.918	8.5		23.6	5.38	2.26	2.01
**Fennel Seed Paste By-Product**										
**Proposed Compound**	**Rt (min)**	**EC**	***m/z* exp.**	**Delta** **(ppm)**	**RDB**	**Ref.**	**Extract (Peak Area × 10^6^)**			
							**SFE-CO_2_**		**UAE**	
							**10% EtOH**	**20% EtOH**	**EtOH**	**EtOH/H_2_O**
**Malic acid**	0.64	C_4_H_5_O_5_	133.0147	4.086	2.5	[52]	1.56	17.0	4.32	50.9
**Quercetin-O-Glucuronide**	6.35	C_21_H_17_O_13_	477.0675	0.034	13.5	[52]	-	20.8	9.61	35.5
**Chlorogenic acid**	5.02	C_16_H_17_O_9_	353.0881	0.551	8.5	[52]	0.05	12.7	6.45	6.87
**Kaempferol-O-Glucuronoside**	6.70	C_21_H_17_O_12_	461.073	0.978	13.5	[53]	-	6.46	3.13	6.45
**2-(Hydroxymethyl)-1,2,3,4-butanetetrol or deoxytetritol**	0.61	C_5_H_11_O_5_	151.06163	2.868	0.5	[53]	2.21	1.21	0.92	0.43
**Casearicoside A**	5.17	C_18_H_25_O_11_	417.1408	1.235	6.5	[54]	11.9	16.7	27.9	2.07
Myristicin *	5.15	C_11_H_13_O_3_	193.08562	−1.558	5.5	[54]	26.4	-	44.6	2.97
2,4-Thujanediol *	6.37	C_10_H_19_O_2_	171.13763	−1.907	1.5	[54]	22.6	-	37.8	3.14
**Isorhamnetin**	8.77	C_16_ H_11_O_7_	315.0513	0.965	11.5	[55]	0.41	0.23	0.39	-
**Naringenin**	8.17	C_15_ H_11_O_5_	271.0613	0.418	10.5	[53]	0.33	0.09	0.29	-
**Rosmarinic acid**	7.56	C_18_ H_15_O_8_	359.0775	0.862	11.5	[55]	0.09	0.38	1.57	-

* Ionization in positive mode.

## Data Availability

Data is contained within the article or supplementary material.

## Supplementary Materials

The following are available online, Table S1: Details for the cold press procedure, Figure S1a: HPLC-UV chromatograms of grape seed paste extracts at 280 nm (black: UAE EtOH, red: UAE EtOH/H2O 1:1 *v/v*, brown: SFE 10% EtOH, purple: SFE 20% EtOH), Figure S1b: HPLC-UV chromatograms of pomegranate seed paste extracts at 280 nm (black: UAE EtOH, red: UAE EtOH/H2O 1:1 *v/v*, brown: SFE 10% EtOH, purple: SFE 20% EtOH), Figure S1c: HPLC-UV chromatograms of hemp seed paste extracts at 280 nm (black: UAE EtOH, red: UAE EtOH/H2O 1:1 *v/v*, brown: SFE 10% EtOH, purple: SFE 20% EtOH), Figure S1d: HPLC-UV chromatograms of fennel seed paste extracts at 280 nm (black: UAE EtOH, red: UAE EtOH/H2O 1:1 *v/v*, brown: SFE 10% EtOH, purple: SFE 20% EtOH), Figure S2a: UPLC-HRMS/MS-ESI(-) analysis of grape seed paste extracts. A: BP-TIC of SFE-CO2 + 10% EtOH extract, B: BP-TIC of SFE-CO2 + 20% EtOH extract, C: UAE-EtOH extract and D: UAE-EtOH/H2O 1:1 *v/v* extract, Figure S2b: UPLC-HRMS/MS-ESI(-) analysis of pomegranate seed paste extracts. A: BP-TIC of SFE-CO2 + 10% EtOH extract, B: BP-TIC of SFE-CO2 + 20% EtOH extract, C: UAE-EtOH extract and D: UAE-EtOH/H2O 1:1 *v/v* extract, Figure S2c: UPLC-HRMS/MS-ESI(-) analysis of hemp seed paste extracts. A: BP-TIC of SFE-CO2 + 10% EtOH extract, B: BP-TIC of SFE-CO2 + 20% EtOH extract, C: UAE-EtOH extract and D: UAE-EtOH/H2O 1:1 *v/v* extract, Figure S2d: UPLC-HRMS/MS-ESI(-) analysis of fennel seed paste extracts. A: BP-TIC of SFE-CO2 + 10% EtOH extract, B: BP-TIC of SFE-CO2 + 20% EtOH extract, C: UAE-EtOH extract and D: UAE-EtOH/H2O 1:1 *v/v* extract, Figure S3: Results of TPC assay. The results are presented as mg of gallic acid equivalent (GAE) per g for the produced extracts from grape seeds, pomegranate seeds, hemp seed and fennel seeds at the concentration of 10mg/mL. Each extraction method is depicted on the Y axis, Figure S4: Results of the DPPH radical scavenging activity assay. The percentage of DPPH radical scavenging activity (%) is presented for the produced extracts from (A) grape seeds, (B) pomegranate seeds, (C) hemp seed and (D) fennel seeds. Gallic acid (GA) was used as positive control.

## Sample Availability

Samples of the compounds are available from the authors.
