# Supplementary material for: Exploitation of Vitis vinifera, Foeniculum vulgare, Cannabis sativa and Punica granatum By-Product Seeds as Dermo-Cosmetic Agents
Source: Molecules. 2021 Jan 31;26(3):731. doi: 10.3390/molecules26030731 (PMC7866782; doi:10.3390/molecules26030731)
Supplement: Supplementary file 1 [file molecules-26-00731-s001.pdf]

Supplementary Materials

Exploitation of *Vitis vinifera, Foeniculum vulgare, Cannabis sativa* and *Punica granatum* by-product seeds as dermo-cosmetic agents.

Dimitris Michailidis[1], Apostolis Angelis[1], Panagiota Efstathia Nikolaou[2], Sofia Mitakou[1], Alexios Léandros Skaltsounis[1]*

[1] *Faculty of Pharmacy, Department of Pharmacognosy and Natural Products Chemistry, National and Kapodistrian University of Athens, Athens, 15772, Greece*

[2] *Faculty of Pharmacy, Department of Pharmacology, National and Kapodistrian University of Athens, 15771 Athens, Greece*

*Corresponding author; E-mail: skaltsounis@pharm.uoa.gr; Tel: 0030 2107274598

Supplementary Materials:

Supplementary table

Table S1: Details for the cold press procedure

Plant	***Raw material***	***Treated amound***	***Treatment speed***	***Prodused oil***	***% of oil production (v/w of seed)***	***By-product seed paste***	***% By-product production (w/w of seed)***
Vitis vinifera	***Grape seed*** *(GrpS)*	*30 Kg*	*15 kg/h*	*2.1 L*	*7%*	*28.3 Kg*	*94.2%*
Punica granatum	***Pomegranate seed*** *(PmgS)*	*25Kg*	*15 kg/h*	*1.75 L*	*7%*	*23.7 Kg*	*94.9%*
Cannabis sativa	***Hemp seed*** *(HmpS)*	*20 Kg*	*25 kg/h*	*3.8 L*	*19%*	*16.8 Kg*	*84.1%*
Foeniculum vulgare	***Fennel seed*** *(FnnS)*	*40 Kg*	*25 kg/h*	*0.6 L*	*1.5%*	*39.4 Kg*	*98.6%*

Supplementary Materials

Supplementary figures

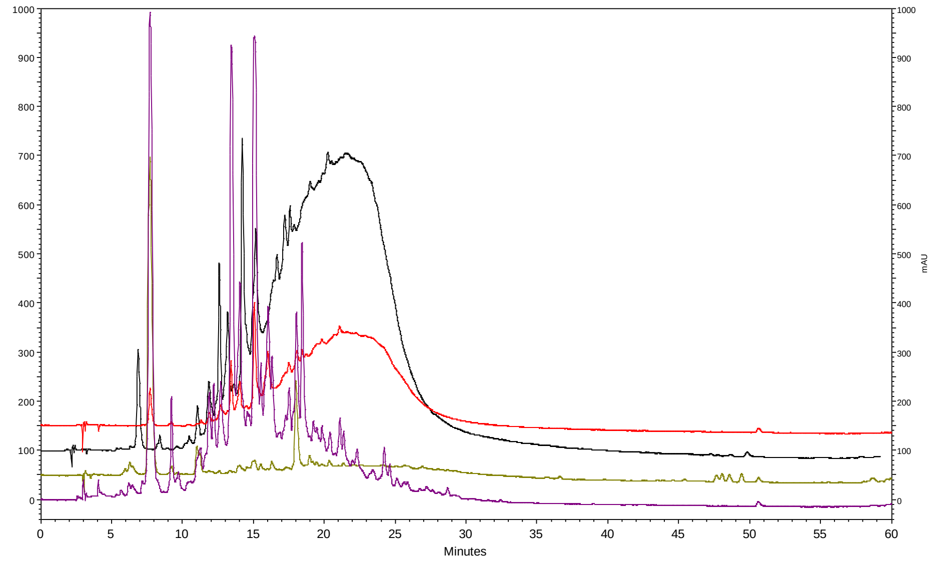

Figure S1a: HPLC-UV chromatograms of grape seed paste extracts at 280 nm (black: UAE EtOH, red: UAE EtOH/H2O 1:1 v/v, brown: SFE 10% EtOH, purple: SFE 20% EtOH).

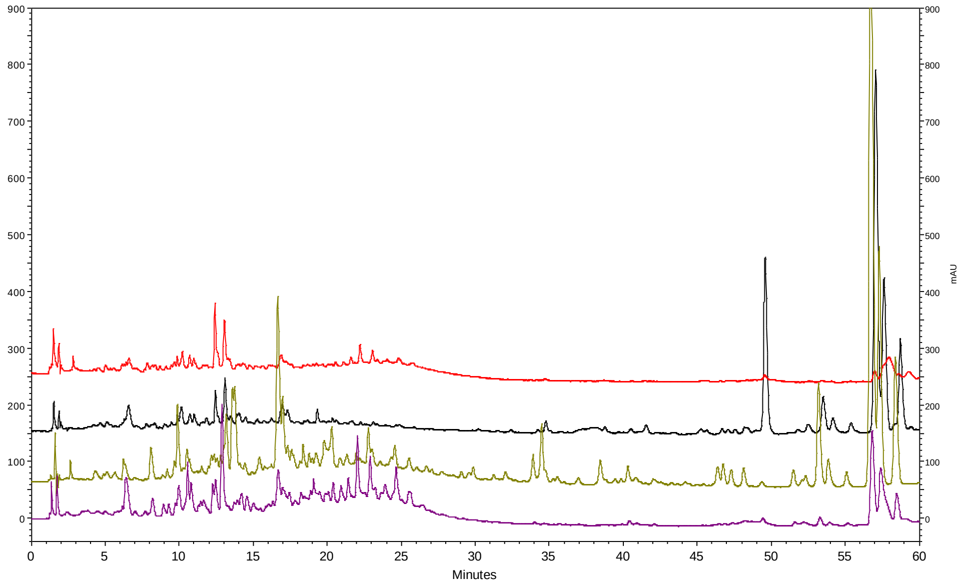

Figure S1b: HPLC-UV chromatograms of pomegranate seed paste extracts at 280 nm (black: UAE EtOH, red: UAE EtOH/H2O 1:1 v/v, brown: SFE 10% EtOH, purple: SFE 20% EtOH).

Supplementary Materials

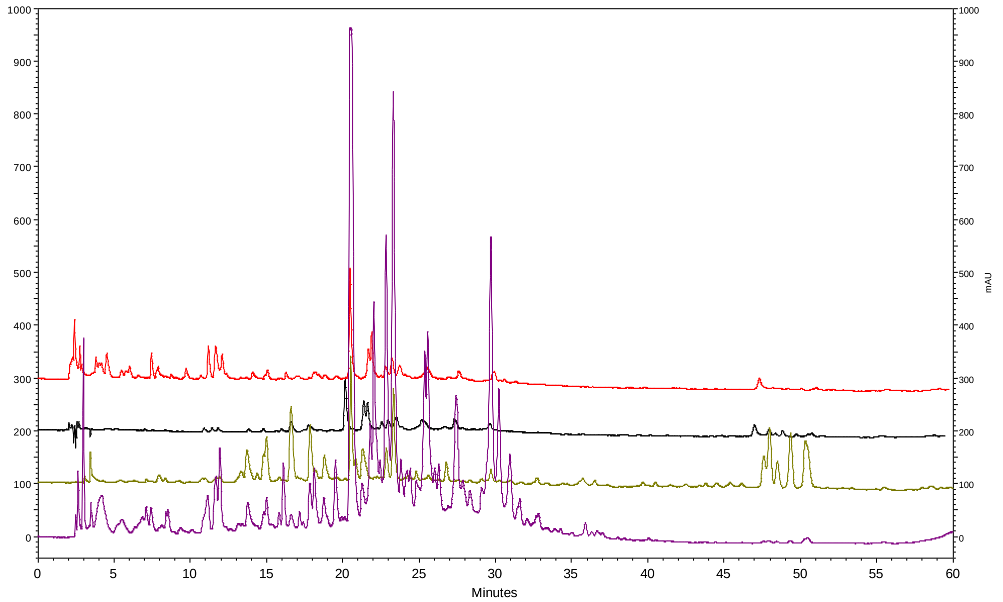

Figure S1c: HPLC-UV chromatograms of hemp seed paste extracts at 280 nm (black: UAE EtOH, red: UAE EtOH/H_2O 1:1 v/v, brown: SFE 10% EtOH, purple: SFE 20% EtOH)**.**

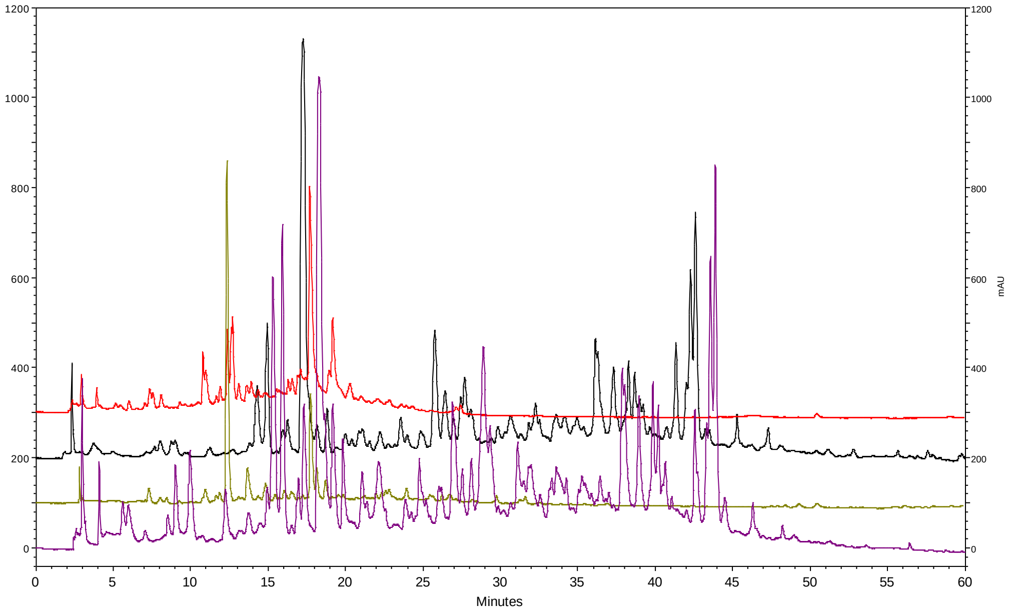

Figure S1d: HPLC-UV chromatograms of fennel seed paste extracts at 280 nm (black: UAE EtOH, red: UAE EtOH/H2O 1:1 v/v, brown: SFE 10% EtOH, purple: SFE 20% EtOH).

Supplementary Materials

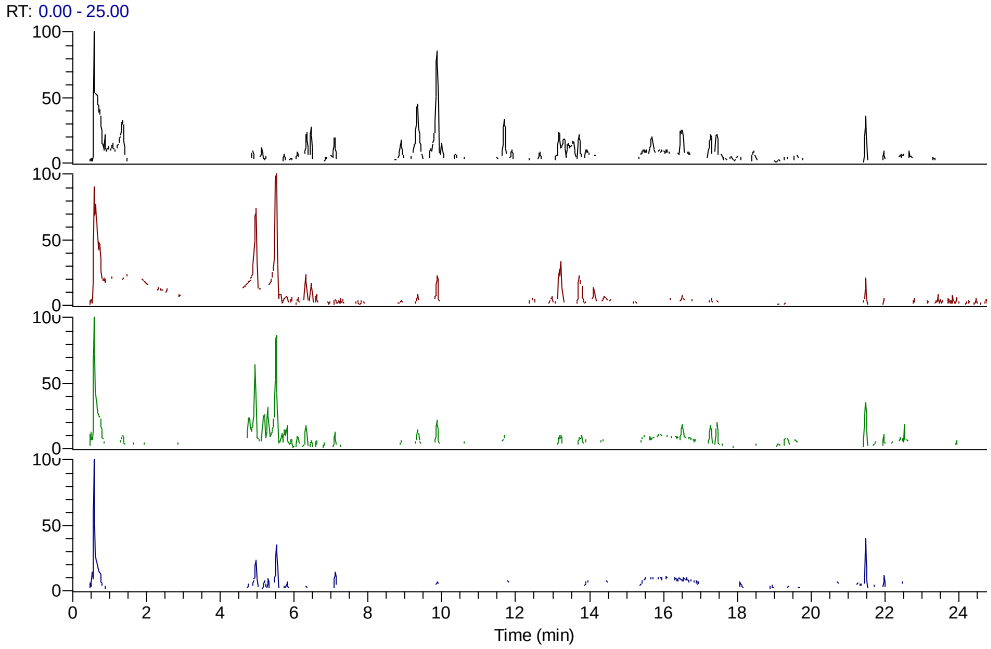

Figure S2a: UPLC-HRMS/MS-ESI(-) analysis of grape seed paste extracts. A: BP-TIC of SFE-CO_2 + 10% EtOH extract, B: BP-TIC of SFE-CO_2 + 20% EtOH extract, C: UAE-EtOH extract and D: UAE-EtOH/H2O 1:1 v/v extract.

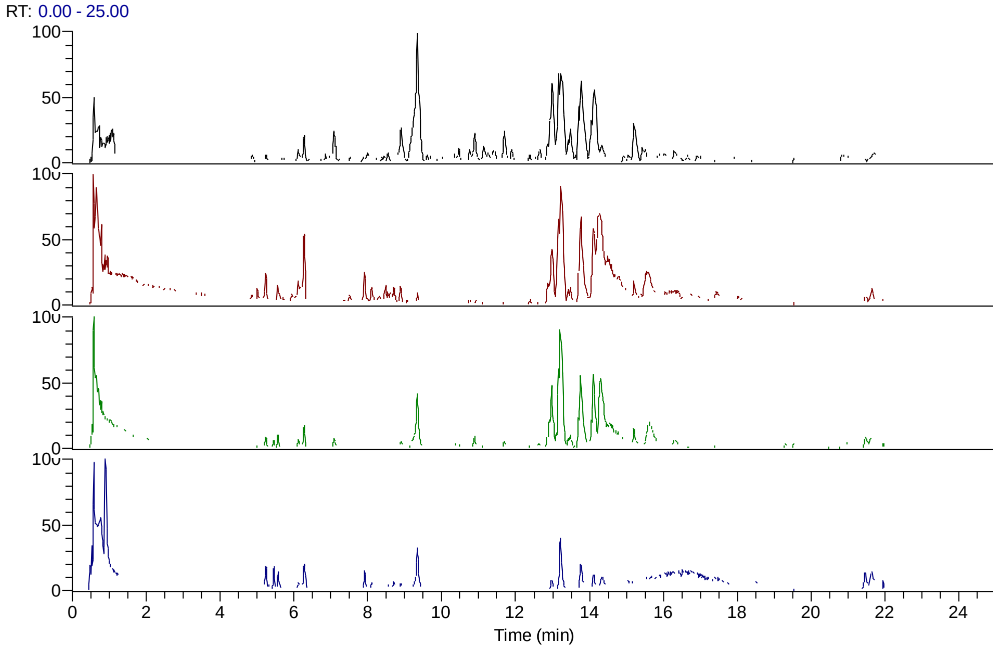

Figure S2b: UPLC-HRMS/MS-ESI(-) analysis of pomegranate seed paste extracts. A: BP-TIC of SFE-CO_2 + 10% EtOH extract, B: BP-TIC of SFE-CO_2 + 20% EtOH extract, C: UAE-EtOH extract and D: UAE-EtOH/H2O 1:1 v/v extract.

Supplementary Materials

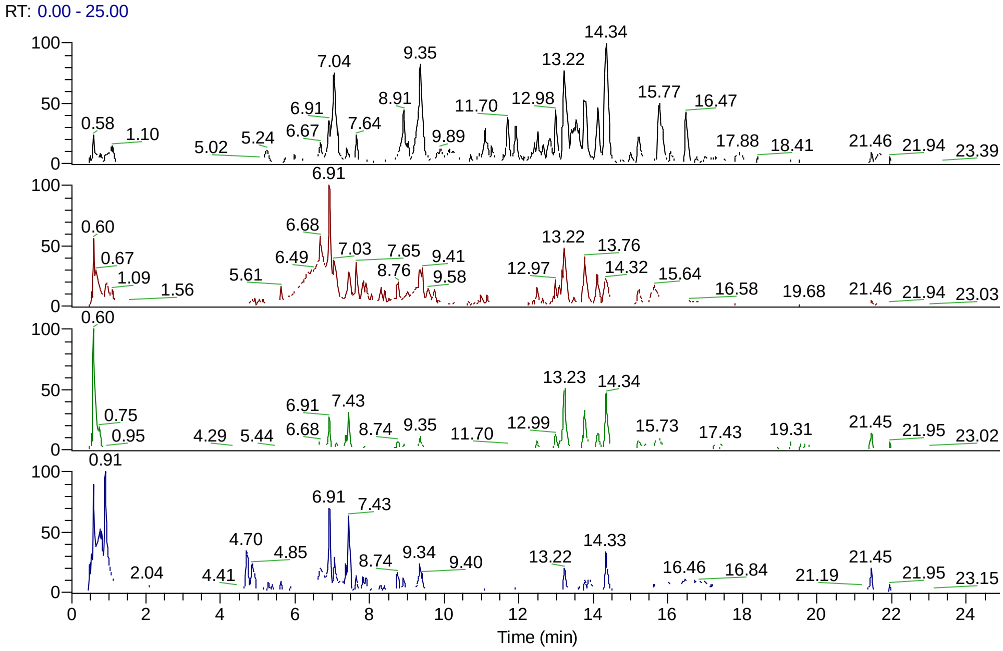

Figure S2c: UPLC-HRMS/MS-ESI(-) analysis of hemp seed paste extracts. A: BP-TIC of SFE-CO_2 + 10% EtOH extract, B: BP-TIC of SFE-CO_2 + 20% EtOH extract, C: UAE-EtOH extract and D: UAE-EtOH/H2O 1:1 v/v extract.

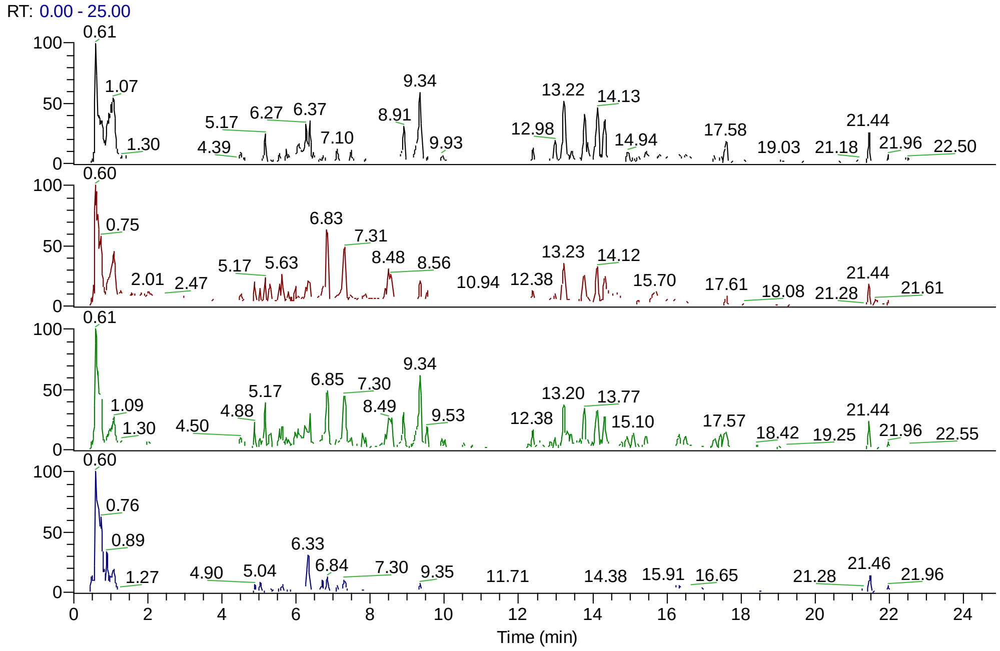

Figure S2d: UPLC-HRMS/MS-ESI(-) analysis of fennel seed paste extracts. A: BP-TIC of SFE-CO_2 + 10% EtOH extract, B: BP-TIC of SFE-CO_2 + 20% EtOH extract, C: UAE-EtOH extract and D: UAE-EtOH/H2O 1:1 v/v extract.

Supplementary Materials

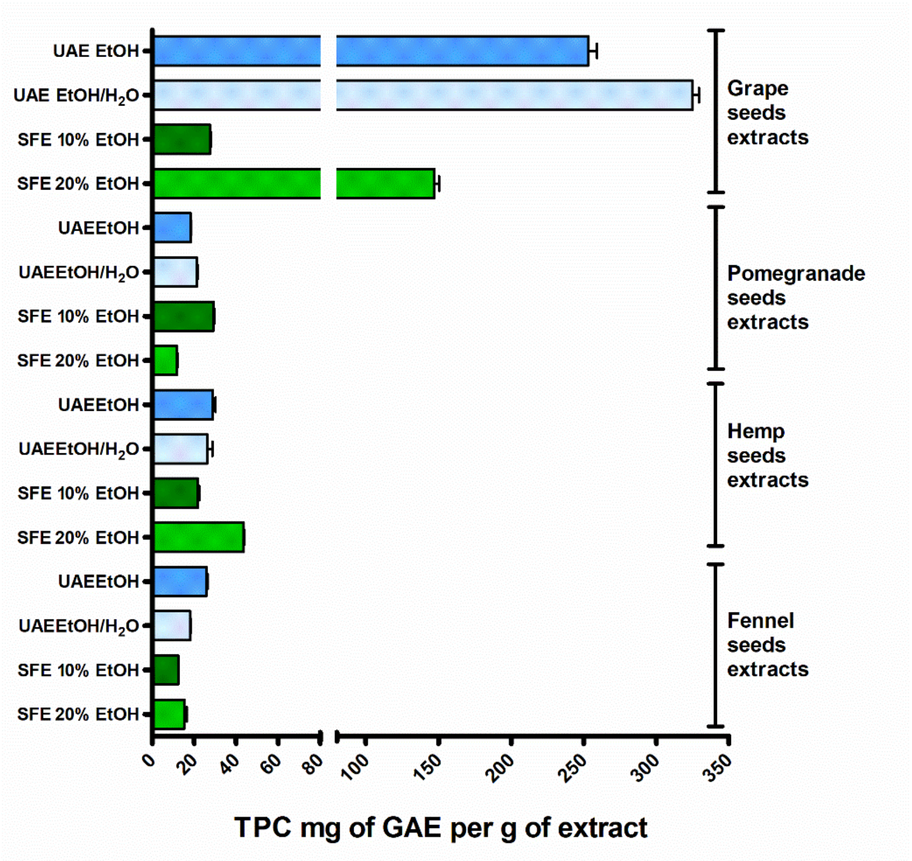

Figure S3: Results of TPC assay. The results are presented as mg of gallic acid equivalent (GAE) per g for the produced extracts from grape seeds, pomegranate seeds, hemp seed and fennel seeds at the concentration of 10mg/mL. Each extraction method is depicted on the Y axis.

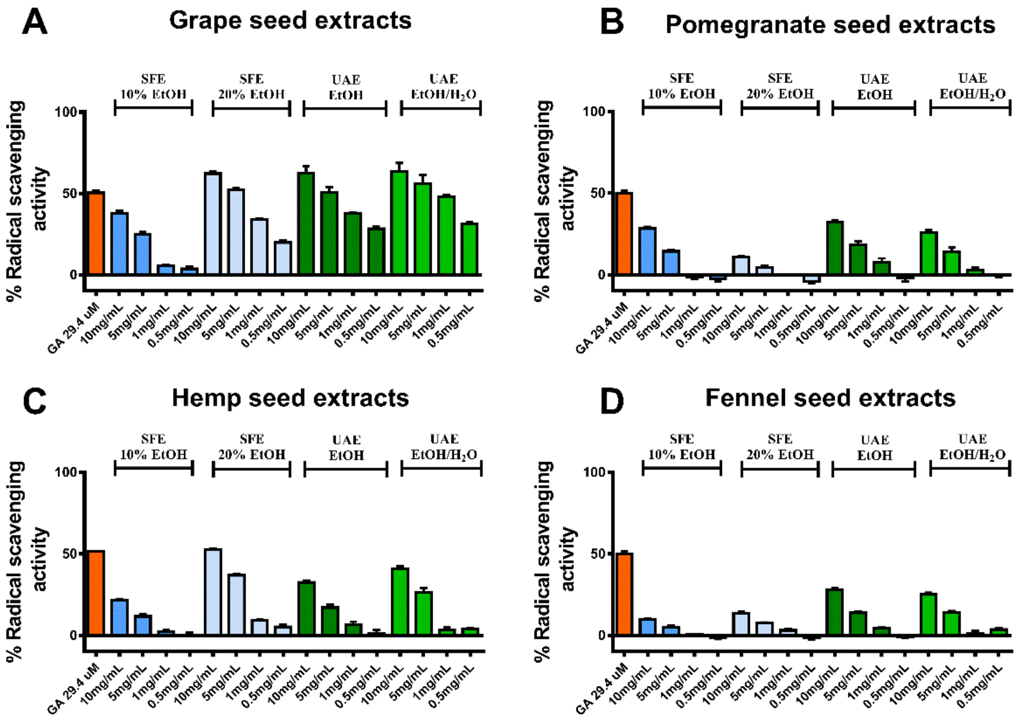

Supplementary Materials

Figure S4: Results of the DPPH radical scavenging activity assay. The percentage of DPPH radical scavenging activity (%) is presented for the produced extracts from (A) grape seeds, (B) pomegranate seeds, (C) hemp seed and (D) fennel seeds. Gallic acid (GA) was used as positive control.
